# Comparison the Emergency Severity Index and Manchester Triage System in Trauma Patients

**DOI:** 10.30476/BEAT.2022.92297.1302

**Published:** 2022-04

**Authors:** Hossein Zakeri, Lahya Afshari Saleh, Shabnam Niroumand, Maryam Ziadi-Lotfabadi

**Affiliations:** 1 Department of Emergency Medicine, Mashhad University of Medical Sciences, Mashhad, Iran; 2 Department of Occupational Medicine, Division of Sleep Medicine, Psychiatry and Behavioral Sciences Research Center, Mashhad University of Medical Sciences, Mashhad, Iran; 3 Department of Community Medicine, Faculty of Medicine, Mashhad University of Medical Sciences, Mashhad, Iran

**Keywords:** Trauma Severity Indexes, Patient outcome, Triage, Emergency medicine

## Abstract

**Objective:**

To compare the emergency severity index (ESI) and Manchester triage system (MTS) in trauma patients.

**Methods:**

This cross-sectional study was conducted by census method in Hasheminejad hospital during 2019. Patients referred to a trauma center triaged by five trained triage nurses based on ESI and MTS. Outcomes were considered as length of stay at the emergency department, admission to the other sectors and discharge or leave the hospital. Information from the triage form, nursing registry office and hospital registry system were extracted and analyzed by SPSS software.

**Results:**

Totally 447 and 468 patients triaged with the ESI and MTS were included, respectively. Seventy percent of patients triaged with ESI and 34% with MTS were placed in level 3 or the yellow group (equivalent group 3 triage). The hospitalization rate is approximately equal at each triage level in the both systems. The mortality rate in both groups was 0%. Mean length of stay was significantly lower in the MTS group compared to ESI in the emergency department (*p*<0.05).

**Conclusion:**

Using of ESI triage in the trauma center causes to arrive more patients to the emergency department instead of the fast track and leads to waste the time and energy of staff’. However, further studies are needed to prove this result.

## Introduction

Traumatic injury is one of the leading causes of mortality and morbidity across the globe [1]. Severe trauma assumes critical importance in the health domain since trauma-related disability negatively affects the role of trauma patients in the family and society. Therefore, the management of these patients within the first hour after the injury is of utmost importance. Triage is defined as prioritizing or sorting the patients to receive care and treatment due to a shortage of the necessary resources in the emergency department. Triage systems are designed to maintain human life and health based on the concept of equitable use of resources.

The growing imbalance between needs and outcomes has increased the need for emergency department triage of patients [2-4]. Triage is the initial assessment of patients in the emergency department in order to sort them to receive diagnostic and therapeutic measures. It primarily aims to diagnose patients who are in urgent need of care and cannot wait to receive diagnostic and therapeutic measures [5, 6]. The most commonly used triage systems in developed countries are the Manchester Triage System (MTS) and the Emergency Severity Index (ESI). 

There are many advantages in using the Manchester triage system, including easy use and learning, as well as high-speed application and implementation [4]. This system also specifies physician visits and patient waiting times [7-9]. It also prioritizes patients based on existing signs and symptoms without any hypothesis about the underlying diagnosis, based on 53 flowcharts [10-12]. The MTS is organized by the Manchester group and includes 53 flowcharts based on patient complaints. In each flowchart, determinants or variables are designed based on the patient’s problems and allocate the patient to one of the following five groups:

Red group (urgently need physician visit), orange group (can wait 10 minutes), yellow group (can wait 1 hour), green group (can wait 2 hours), blue group (can wait 4 hours) [13-15]. Unlike the MTS, the ESI has a flowchart and ranks a patient’s condition based on a scale of 1-5: 1= requires immediate intervention to survive, 2= high-risk condition, 3= patient needs two or more resources, 4= patient needs one source, and 5= no resource needed [16-18]. Resources include laboratory procedures, radiology, intravenous fluids, specific consultations, simple or complex procedures, as well as intramuscular, intravenous, and inhaled medications [18, 19].

The ESI triage system is currently used in all emergency departments of medical centers in Iran. In light of the aforementioned issues, the present study aimed to compare the outcomes of hospitalization and death of trauma patients using ESI and MTS.

## Materials and Methods

This cross-sectional study was conducted by census method in the emergency department of Shahid Hasheminejad Research and Training Center from January 20 to March 20, 2019. Shahid Hasheminejad hospital is a general referral teaching hospital of Mashhad university of medical sciences with 320 beds in 22 hospital section located in northeast of Mashhad city. All trauma patients who referred to Shahid Hasheminejad trauma center in the time of the project, were enrolled in the study. A total of 915 trauma patients were triaged by five trained triage nurses with Severity Index and Manchester Triage Systems. The current research was approved by the ethics committee of Mashhad University of Medical Sciences (IR.MUMS.MEDICAL.REC.1398.316).

*Methods and Steps Implementation of Emergency Severity Index*

Currently, trauma patients who are referred to the accident and emergency department of this hospital are triaged by five trained triage nurses using the ESI system in both days and night shifts. Firstly, triage nurses were retrained on the ESI triage by researchers to reduce the possibility of errors. Since ESI triage is performed routinely in the hospital, all referred patients to the emergency department and meeting the inclusion criteria were included in the study in order to collect samples of the ESI system from the commencement of the study (January 20, 2019) until data saturation.

In ESI as a five-level triage method, patients are divided on the basis of disease severity and the expected needed resources. The first criterion is determined by the presence or absence of life-threatening factors and organs, as well as serious symptoms and vital signs. Moreover, the second one is determined based on the nurse’s experience and comparing the patient with similar cases [18, 19].

*Methods and Steps Implementation of Manchester Triage System*

For MTS triage, first, five skilled emergency nurses without any experience of triage working were selected to prevent any bias and perform MTS triage. These nurses received 4 hours of theory training and a 4-hours practice session according to the educational standards. Thereafter, they were evaluated on the correct performance of MTS using several examples in the training sessions. The minimum sample size for determining the Kappa coefficient was calculated based on the assumption of minimum agreement between observer (30%) and minimum relative error of 20% was 278 patients. Therefore, the calculated Kappa for the degree of agreement between observers was 81% and 95% confidence interval were 0.79-0.83. To collect the samples of MTS since February 20, 2019, all patients who were referred to the emergency department were triaged using the MTS and transferred to the emergency department in coordination with hospital officials.

The MTS consists of 53 flowcharts designed based on the patient’s complaint [14, 15]. Prior to the commencement of the study, the Manchester group was requested to introduce the flowcharts related to the trauma center. Therefore, out of 53 flowcharts in the trauma center, 11 trauma-related flowcharts were selected. The results of patient’s care were retrieved from the nursing registry, hospitalization records, and hospital registration system as follows:

- Hospitalization (admission to ICU-ward), discharge, emergency department length of stay, death in the first 24 hours of hospitalization

The exclusion criteria entailed incomplete medical records, transfer of patients to another medical center in the first 24 hours, and death the patient upon admission to the emergency department. It is noteworthy that in the study center, patients with triage categories of 1, 2, and 3 are transferred to the emergency department, while those assigned to levels 4 and 5 are sorted into the fast track system.

**Fig. 1:**
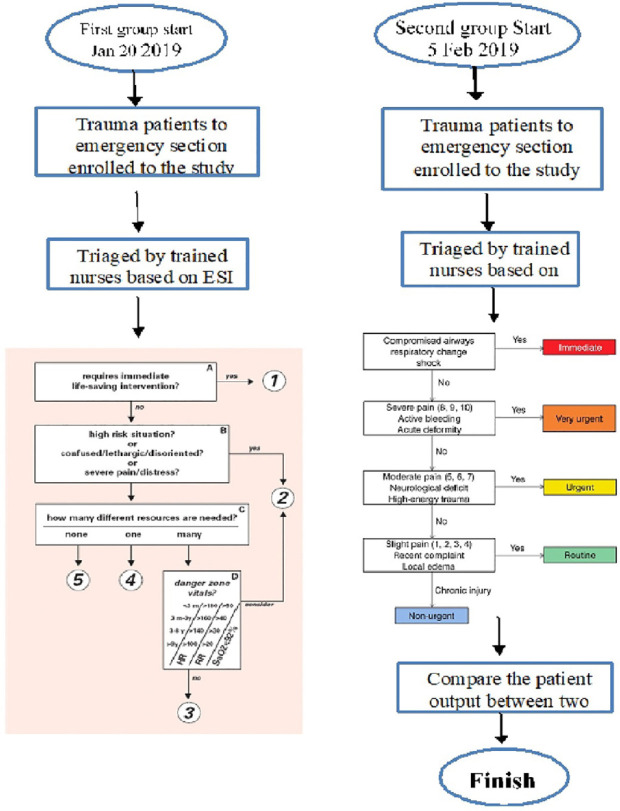
The schematic study flow chart

Figure 1 demonstrated the study flow diagram.

## Results

The present study was conducted on a total of 915 patients referring to the emergency department of Hasheminejad hospital, Mashhad, over one-month period for each group (emergency severity index and Manchester triage). For the purpose of the study, 447 (48.9%) and 468 (51.1%) patients were triaged using ESI and MTS.

*Demographic Characteristics of Patients*

The minimum and maximum age scores of patients in the ESI group were reported as 1 and 90 years, and in the MTS group, the youngest and oldest patients were 1 and 97 years old, respectively. The mean age scores of the patients in the ESI and MTS groups were 30 and 28.5 years, respectively. Furthermore, 66.8% of patients were men and 33.2% were women, and the patients in the two groups did not differ significantly in terms of gender and age (*p*>0.05) (Table 1).

**Table 1:** Demographic characteristics of patients triaged with emergency severity index and the Manchester triage system

***p*** **-value**	**Manchester** **N=468** **N (%)**	**ESI** ^a^ **N=447** **N (%)**	**Demographic characteristics**
0.2	302 (64.5%)	309 (69.1%)	Men
	166 (35.5%)	138 (30.9%)	Women
0.05	28.5 (18.6)	30.39 (18.37)	Age (mean (SD^d^))
	97-1	90-1	Min^b^-Max^c^

^a^ESI: The emergency severity index; ^b^Min: Minimum; ^c^Max: Maximum; ^d^SD: Standard Deviation

*Frequency of Patients by Level/Category*

**Fig. 2:**
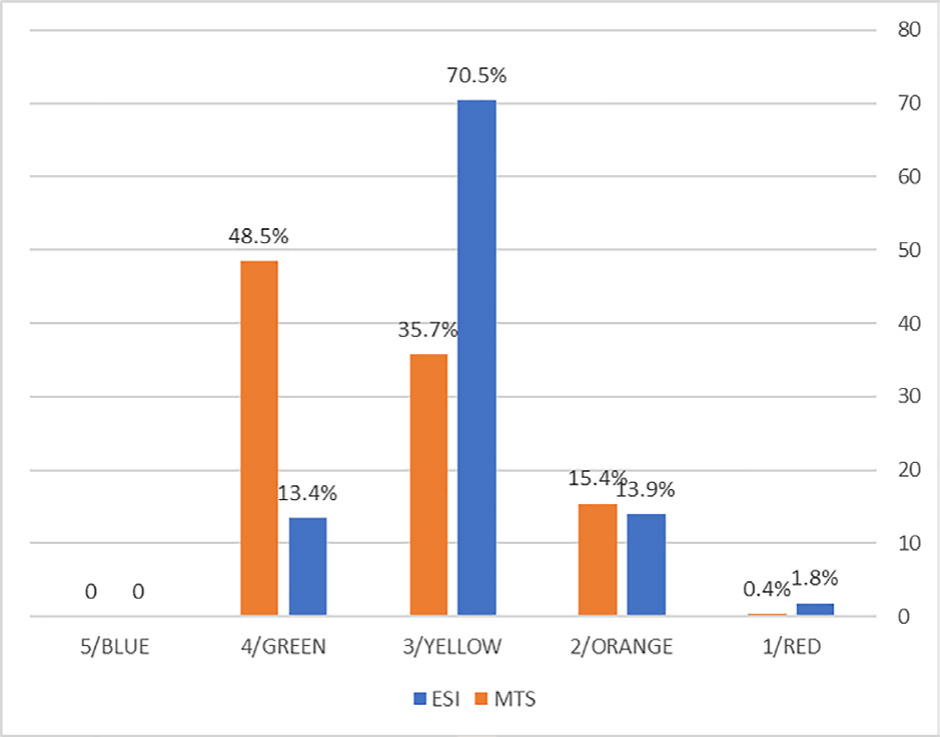
Frequency of patients by level/category in emergency severity index and Manchester triage system

In ESI group, 8 (1.8%), 62 (13.9%), 315 (70.5%), and 62 (13.9%) cases were triaged into levels 1, 2, 3, and 4. None of the patients were assigned to level 5. On the other hand, in the MTS group, 2 (0.4%), 72 (15.4%), 167 (35.7%), and 227 (48.5%) patients were allocated to the red, orange, yellow, and green categories, and none of them were assigned to the blue category (Figure 2).

As illustrated by the results of the study, the ESI system assigned the majority of patients to level 3; that is to explain, about 70% and 14% of cases were sored into levels 3 and 4. Nonetheless, based on MTS, most patients were placed in the green category, therefore, 35% of patients were assigned to the yellow category and 48% of cases fell in the green category.

*Patient Outcome*

**Fig. 3.**
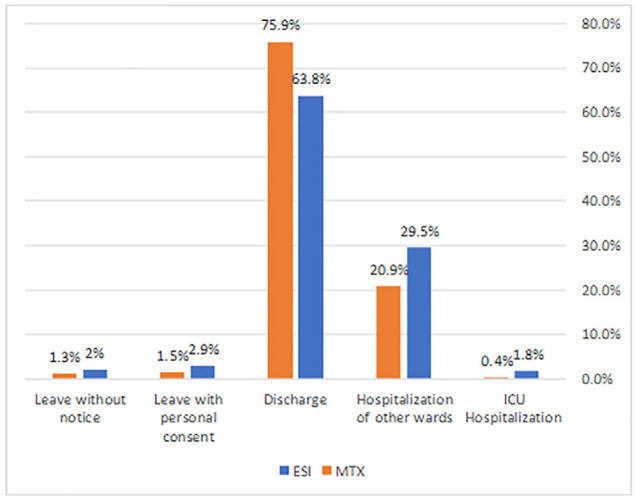
Patient outcomes by emergency severity index and Manchester triage system (in percent)

In total, out of 915 patients, 26% were hospitalized and 73.2% were discharged, and no deaths were reported during the first 24 hours of hospitalization (Figure 3).

In order to compare the efficiency of the two systems in predicting the rate of hospitalization according to the severity of the disease, we calculated the percentage of hospitalized patients in each level/category of triage to the total number of cases triaged to the same level/category. Hospitalization rates in ESI triage levels 3 and 4 were obtained at 26% and 40%, while 31% and 9% of cases in the yellow and green categories of MTS were hospitalized (Table 2).

**Table 2:** Comparison of hospitalization by triage level to total hospitalization in the same triage

**Manchester** **N=100** **N (%) **	**ESI** ^a^ **N=140** **N (%)**	**Level/Category**
1 (1%)	7 (5%)	1/Red
26 (26%)	26 (18.6%)	2/Orange
52 (52%)	82 (58.6%)	3/Yellow
21 (21%)	25 (17.9%)	4/Green

^a^ESI: The emergency severity index

We also calculated the percentage of patients admitted to each triage level/category to the total number of cases triaged to the same level/category. Hospitalization rates in ESI triage levels 3 and 4 were obtained at 26% and 40%. On the other hand, 31% and 9% of patients in the yellow and green categories of MTS were hospitalized. We separately analyzed the percentage of hospitalized people in each level/category to the total number of patients triaged to the same level/category in Table 3.

**Table 3:** Percentage of admitted patients in each level/category to the total number of cases triaged to the same level/category

**Manchester** **N (%) **	**ESI** ^a^ **N (%)**	**Level/Category**
1 (50%)	7 (87.5%)	1/Red
26 (36.11%)	26 (41.93%)	2/Orange
52 (31.13%)	82 (26.03%)	3/Yellow
21 (9.25%)	25 (40.32%)	4/Green

^a^ESI: The emergency severity index

The results of the study demonstrated that the ESI triage system assigned 70% and 13% of patients to levels 3 and 4 with hospitalization rates of 26% and 40%, respectively. On the other hand, MTS allocated 35% and 48% of patients to the yellow and green categories with hospitalization rates of 31% and 9%, respectively. As presented in Table 2, 50% of patients assigned to the red category in MTS were hospitalized. Out of the two cases classified as red, one was hospitalized, and one was discharged. The hospitalization rate cannot be analyzed in this category due to the small number of cases.

In the ESI triage group, the minimum and maximum scores of emergency department length of stay were reported as 10 and 630 minutes. On the other hand, in the MTS group, these minimum and maximum times were reported as 5 and 710 minutes. The mean length of stay in the emergency department in the ESI triage system and the MTS has a significant relationship with the triage level/category (except for the patients in level 1/red category) (*p*<0.05). Emergency department length of stay is shortened as the level/category of triage increases and the severity of disease decreases (Table 4).

**Table 4:** Mean length of emergency department stay in emergency severity index and Manchester triage system by triage level/category

***p*** **-value**	**MTS** ^b^ **Mean (SD)**	**ESI** ^a^ **Mean (SD)**	**Level/Category**
0.5	220 (99)	206 (67)	1/Red
0.04	170 (107)	110 (209)	2/Orange
0.01	123 (88)	146 (103)	3/Yellow
<0.001	93 (55)	161 (83)	4/Green

^a^ESI: The emergency severity index; ^b^MTS: The Manchester Triage System

## Discussion

The present study compared the results of two triage systems, namely ESI and MTS in trauma patients. Regarding gender, 66.8% of cases were men, and 33.2% were women. In other studies, performed at the trauma center, the percentage of men patients was higher than women [5, 20]. As mentioned earlier, 1.8% of the cases were allocated to ESI level 1, and 14% were sorted into level 2, and 41% of them were hospitalized. According to a study carried out by Rahmani *et al*., [20] 1-3% and 20-30% of patients were assigned to ESI levels 1 and 2, and 50-60% of them were hospitalized.

Along the same lines, in a study conducted on trauma patients by Chi *et al*., [5] the hospitalization rates at ESI levels 2 and 3 were reported as 45.6% and 28.3%, respectively. These findings are comparable to the results of our study on trauma patients (41% and 26% at the same levels). In our study, the mortality rate within the first 24 hours of hospitalization was obtained at 0; moreover, hospitalization and discharge rates were 26%, and 69.9%, respectively. These results are comparable to those reported by Chi *et al*., [5] who indicated mortality, hospitalization, and discharge rates of 0.4%, 20.8%, and 71.7%, respectively.

On the other hand, in other study [20], 30-40% of patients referred to the emergency department were assigned to ESI level 3, and 20-35% of cases were triaged to levels 4 and 5. These values in the current study were calculated at 70% and 14%, respectively. Since our study was merely performed on trauma patients, the cases were seemingly triaged to level 3 ESI, instead of level 4 due to the need for x-rays and sutures. In our opinion, ESI triage alone is incapable of distinguishing between levels 3 and 4 and poses a challenge to the patient care process. The Emergency Severity Index Version 4 Triage Algorithm also emphasizes that in trauma centers, ESI triage and trauma response level should be calculated separately and the patient should be evaluated based on the result of both.

Based on the obtained results, 15.6% of cases in the ESI triage group were sorted to levels 1 and 2. In the MTS group, 15.8% of cases were triaged into the red and orange categories. As demonstrated by the results of the study, the difference between these two triage systems lies at level 3/yellow category and level 4/green category. The results of the study also indicated that the ESI triage system allocated 70% of patients to level 3, while 26% of them were hospitalized. On the other hand, in the MST group, 35% of cases were assigned to the yellow category, and 31% of them were hospitalized.

A comparison of these two values demonstrates that the ESI triage system sorted the majority of patients to level 3. As mentioned earlier, in the ESI system, if the patient is not allocated to levels 1 and 2, the nurses triage the patient to level 3 or 4 based on the number of needed resources [14-17]. Therefore, since trauma patients need X-rays, CT scans, or sutures, the patient is triaged to level 3 regardless the severity of the disease [20]. From our perspective, the disadvantage of the ESI triage system is the allocation of patients with levels 3 and 4.

The mean scores of emergency department length of stay in ESI and MTS groups were obtained at 157 and 115 minutes, respectively, demonstrating a significant difference (*p*<0.001). The difference between this variable in the two groups can be described to the different distribution of the triage level in the two triage systems. The majorities of patients are triaged to ESI level 3 and require inpatient care, rather than outpatient services.

Therefore, level 3 patients had to wait 42 minutes longer in the emergency department to be visited, resulting in congestion during peak busy periods of patient attendance at the emergency department. Therefore, it is recommended that future studies need to be performed in other trauma centers with larger sample size. In ESI triage, the vital signs were used to categorize patients include pulse rate, respiration rate, oxygen saturation for all age groups, and body temperature in all children less than 3 years of age.

In Manchester triage, body temperature and oxygen saturation are measured according to the type of flowchart. In this triage system, the patient is evaluated for inadequate and ineffective breathing or acute dyspnea (shortness of breath), which makes more sense than assessing the number of breaths in the ESI triage in the trauma center. In their study, Farrokhnia *et al*., [21] conducted a systematic review of triage tools in the emergency department and their contents. The results of the referred study showed that the number of breaths was only mentioned in one study as the important mortality predictor. Moreover, they found that the assessment of blood pressure and body temperature were not recognized as important factors associated with mortality in emergency department.

 As evidenced by the results of the present study, the use of MTS in trauma patients significantly reduces the length of emergency department stay and saves hospital resources (manpower and equipment). Nevertheless, since this study has not yet been performed in trauma patients, the necessary decisions need further studies.

*Strengths and Weaknesses*

This is the first study comparing the two triage systems among trauma patients. The two group nurses were exactly trained and assessed in terms of the task correctness by researcher. Moreover, they were separated and not aware of the other triage system. The study carried out at the same season and hospital in order to minimize the role of other covariates. 

Although the study had some weak points. MTS nurses were less experienced than the other group. In addition, no mortality (as the main patient outcome) was recorded in the present study and the distribution of patients with different triage levels was not homogeneous. The study was conducted in one hospital due to the difficulty of administrative accord.

## Declaration

### Ethics approval and consent to participate:

The current research was approved by the ethics committee of Mashhad University of Medical Sciences (IR.MUMS.MEDICAL.REC.1398.316).

### Consent for publication:

The authors express their consent to the publication of the article.

### Conflict of Interest:

None declared.

### Funding:

This study was funded by Mashhad University of Medical Sciences, Mashhad, Iran.

### Authors’ contributions:

HZ, SN and SMM contributed in study design, variable selection, and revise of the study. MZL contributed in patient sampling, checklist Design and followed the patients and their outcomes as an assistant. SN and MZL contributed in statistical analysis and study revise. All of authors read and approved the final manuscript.

### Acknowledgement:

The authors are grateful to the participants, their parents, and hospital staff of Shahid Hasheminejad hospital, Mashhad, Iran, for their cooperation.
